# Atlanta Medical College

**Published:** 1866-05

**Authors:** 


### EDITORIAL AND MISCELLANEOUS.

---

### ATLANTA MEDICAL COLLEGE.

This institution is about entering upon its eighth regular Summer Course of Lectures. The building, up to a few days since, has been occupied as a hospital since 1862. The Confederate and Federal armies, accordingly as one or the other of these authorities were in possession of the city, found here comfortable accommodation for two hundred of their sick and wounded. In order to make it suitable for this purpose, the amphitheatre and chemical lecture room were deprived of the expensive fixtures they contained. Half the building was obtained from the military authorities in November last, and fitted up for the winter session. The library, museum and laboratory, though the subjects of considerable depredations, with a moderate outlay, have been sufficiently replenished for all practical purposes at present. Seats and other fixtures, in all the lecture rooms, have been replaced in exactly the former styles. All other repairs necessary for the comfort and convenience of teacher and student, have been made. Material in abundance, for the practical study of anatomy, and plates, models, wet and dried preparations, specimens, etc., etc., suited to the practical demonstration of the several departments of medical science, are in readiness for the approaching session.

The heavy expenditures necessary thus to supply the appliances, and refit the building, have been made by the sacrifice of personal convenience, that the expectations formed from facilities heretofore afforded by the College, should not be disappointed in the class soon to assemble.

From data in our possession, we conclude the number in attendance this session will far exceed the expectations of the Faculty when the announcement of the Course was published. Indeed, the interest manifested recently would indicate an attendance equal in numbers to an average of the classes in more prosperous times prior to the war.

The Faculty undertake the labors of the Course, commencing first Monday (seveth day) in May, with renewed energy, and with the determination conscientiously to discharge their duty to the student, to the profession and to the public.

That system of instruction thought best to promote a thorough understanding of the principles of medical science, will be pursued. The impressive mode of teaching by daily recapitulations, and by the examinations, of those desiring it, upon the previous lecture, is a plan adopted by the Faculty, which we think cannot be too highly estimated. When a large number of facts is presented to the mind, it is only by repeatedly and impressively calling attention to them that they can be retained.

In the study of Anatomy by demonstrations and dissections, we think the summer gives decided advantages over the winter months. The idea that subjects could not be kept for use during warm weather, upon which depended mainly the opinion that "medicine cannot be successfully taught during the summer," has effectually exploded. It has been practically demonstrated for the last ten years, that subjects properly prepared may be kept for any desirable length of time, and may be used for weeks during the warmest weather without unpleasant odor or softening of the tissues.

By an arrangement effected with the authorities, the freedman's hospital, located conveniently to the College, is made subservient to the interest of the Institution, in the study of clinical medicine. A variety of the ordinary acute and chronic diseases of the country, are made available daily for this purpose. While very large hospitals may give additional advantages to the advanced student, whose course of didactic instruction is concluded, yet to those being taught the fundamental principles of the science of medicine, a hospital of seventy-five patients affords all the material necessary, for the time that can be profitably devoted to clinical study.

All the surgical operations in the private practice of the Professor of Surgery, which can with propriety be performed in the operating room of the College, and all surgical cases in the hospital, will be presented for the benefit of the class.

We know very little of the determinations and movements of sister institutions, South, but we think they are getting under way for a renewal of their labors. Most of them have already held one session since peace was restored. We wish all of them success and prosperity in their laudable efforts to inculcate the prinples of scientific and honorable medicine, and we hope the Faculty of the Atlanta Medical College, as a body, will be of one mind in keeping aloof from petty jealousies, the spirit of dishonorable rivalry and useless contentions. At the same time we shall advocate the unflinching maintenance of those principles acknowledged by medical schools, as the foundation upon which the honor of the profession, and welfare of the community depend. Honest differences of opinion may exist, as to the system of instruction which gives the greatest advantages to those preparing for a place in the profession, but all acknowledge the principle and understand the means of withholding their sanction of dishonor to the profession, and injustice to the public.

Upon medical colleges rest grave responsibilities. They are entrusted with, not only the preparing, but the commissioning duty, and in the perversion of this function, a curse, instead of a blessing, is sent out upon the community.

